# Investigating the incidence of concomitant lower-extremity and pelvic fractures in patients with multiligament knee injuries

**DOI:** 10.1186/s43019-025-00288-z

**Published:** 2025-08-26

**Authors:** Collin D. R. Hunter, Keaton Andra, Joseph Featherall, Benjamin T. Johnson, Patrick E. Greis, Travis G. Maak, Stephen K. Aoki, Antonio Klasan, Justin J. Ernat

**Affiliations:** 1 Department of Orthopaedics, University of Utah, 590 Wakara Way, Salt Lake City, UT 84108 USA; 2 Department of Orthopedics and Traumatology, AUVA UKH Steiermark, Graz, Austria

**Keywords:** Multiligament knee injury, Fracture, Lower extremity, Pelvic fracture

## Abstract

**Background:**

Multiligament knee injuries (MLKIs) often result from high-energy trauma in polytrauma patients. They may coincide with other musculoskeletal injuries, especially fractures of the ipsilateral lower extremity (LE) or pelvis. Understanding these fracture patterns can guide surgical planning and improve patient outcomes. The study aim is to describe the ligamentous injury patterns of combined ipsilateral LE or pelvic fractures with surgically treated MLKIs.

**Methods:**

A retrospective cohort study was conducted from April 2008 to August 2024. Patients who sustained tibial plateau (TP), femoral condyle (FC), fibular, tibial shaft, femoral shaft, or pelvic fractures, concurrent with surgically treated MLKIs, were included. Ligament injuries (anterior cruciate [ACL], posterior cruciate [PCL], medial collateral [MCL], and lateral collateral [LCL]) were categorized by number (≥ 2) and pattern (ACL-based, PCL-based, or bicruciate). Comparisons were made between fracture and non-fracture groups.

**Results:**

Among 211 patients (69% male; mean age 28.3 ± 12.9 years), 36% (75/211) had fractures, with 19% (17/75) requiring operative fixation. TP fractures were the most common (57%), followed by FC (47%) and pelvic fractures (16%). ACL-based injuries (65%) were predominant, while PCL-based injuries were less frequent in fracture patients (4% versus 13% in the non-fracture group). ACL/LCL injuries were significantly more common in the fracture group (29% versus 18%, *p* = 0.049). Two-ligament injuries accounted for 71% (53/75) of fracture cases.

**Conclusions:**

More than one-third of patients with MLKI sustained concomitant LE fractures, with TP fractures occurring most frequently. ACL/LCL and ACL/PCL/LCL patterns showed particularly high fracture rates, whereas PCL-based MLKIs were more common without fractures.

## Background

Multiligament knee injuries (MLKIs) are a rare yet devastating challenge in orthopedic surgery, defined as the disruption of two or more of the cruciate ligaments (anterior cruciate ligament [ACL] and posterior cruciate ligament [PCL]) and collateral ligaments (medial collateral ligament [MCL] and lateral collateral ligament [LCL]) [1–3]. These injuries often result from high-energy trauma, such as motor vehicle collisions, falls from height, or sports-related incidents, and are associated with significant morbidity [4]. The complexity of MLKIs is multifactorial, stemming from the range of potential comorbidities present at the time of injury, including intraarticular and neurovascular compromise, musculoskeletal trauma, and the inherent polytraumatic states involving vital organ injury [5–7]. These injuries are often associated with coincident bony fractures owing to the nature of the high-energy mechanisms involved [8].

 A unique yet understudied aspect of MLKIs is the frequent association with fractures of the lower extremity and pelvis. These fractures add to the already challenging management of MLKIs, potentially complicating both surgical planning and postoperative rehabilitation [3, 7, 9]. While previous research has documented the presence of lower extremity (LE) and pelvic fractures in the context of multiligament knee injuries [10], the interplay between these fractures and associated ligamentous injury patterns remains poorly understood. Existing studies have described the frequency of lower-extremity fractures and associated injuries in MLKIs [10], but there is limited reporting on the relationship between specific ligamentous patterns and fracture types, the distribution of fractures, and the typical number of fractures present in these cases. These findings can help provide insight into the additional injury burden that pelvic and lower-extremity fractures place on patients with concurrent MLKIs. Understanding these relationships is essential to better characterize the complexity of MLKIs and to optimize surgical management and rehabilitation strategies for these patients [11].

 This study aims to describe the LE fracture patterns observed in patients with MLKIs and to compare these patterns with those in MLKIs without associated fractures. Specifically, we seek to evaluate the relationship between fracture presence and various ligamentous injury patterns, categorized by cruciate ligament involvement, number of ligaments injured, and specific combinations of ligamentous injury. We hypothesize that certain ligamentous injury patterns (such as ACL-based injuries) will be more associated with specific fracture patterns than others (e.g., PCL-based injuries). We secondarily hypothesize that high-energy mechanisms will be more associated with a greater number of total fractures.

## Methods

A retrospective cohort study of all patients treated surgically for MLKIs was performed over a 16-year period between April 2008 and November 2024 at a single tertiary academic medical center. MLKIs were defined as injuries requiring surgical reconstruction or repair of a minimum of two ligaments. Fractures were identified as any ipsilateral lower-extremity or pelvic fracture incurred at the same trauma as the MLKI. Contralateral fractures or fractures proximal to the pelvis were excluded. Patients were identified by reviewing operative reports, intake forms, and discharge summaries, and cross-referencing magnetic resonance imaging (MRI) findings. Inclusion criteria included: (1) MLKIs with a minimum of two surgically treated ligaments, (2) availability of preoperative clinical notes documenting the initial encounter and mechanism of injury, and (3) operative reports confirming ligamentous reconstruction or repair. Exclusion criteria included: (1) prior history of MLKI surgery, (2) nonoperative management, and (3) incomplete medical records such as missing discharge summaries to identify additional procedures. All MRI findings were based on formal interpretations by board-certified radiologists at the study institution. Time to surgery is defined as the days from initial injury to surgical fixation of MLKI.

Locations of pelvic and ipsilateral lower-extremity fractures were tabulated depending on the anatomic location: pelvis, femoral shaft (FS), femoral condyle (FC), tibial plateau (TP), tibial shaft (TS), or fibula. Ligamentous injury patterns were stratified and analyzed across four distinct groups: (1) single cruciate with a single collateral ligament injury, including ACL/MCL, ACL/LCL, PCL/MCL, and PCL/LCL injuries; (2) bicruciate MLKIs involving a collateral ligament, comparing ACL/PCL/MCL injuries versus ACL/PCL/LCL injuries versus ACL/PCL/LCL/MCL injuries; (3) those with ACL-based, PCL-based, and bicruciate MLKIs; and (4) the number of ligaments involved, grouped into two-, three-, and four-ligament MLKIs. Pelvic and ipsilateral lower-extremity fractures were tabulated depending on the anatomic location: pelvis (P/MLKI), femoral shaft (FS/MLKI), femoral condyle (FC/MLKI), tibial plateau (TP/MLKI), tibial shaft (TS/MLKI), or fibula (F/MLKI).

Data analysis was performed by importing the dedicated Research Electronic Data Capture (REDCap) database into SPSS version 27 statistical software (IBM Corp., Armonk, NY). Chi-squared and Fisher’s exact test analyses were used to evaluate the rates of fracture location on the basis of ligamentous injury pattern. The normality of continuous variables (age, body mass index [BMI], and days to MLKI surgery) was assessed using the Shapiro–Wilk test. As the data were found to be normally distributed, independent *t*-tests were subsequently used to compare the means between groups.

## Results

From an initial database of 226 patients, 211 patients (146 males and 65 females) met the inclusion criteria, with a mean age of 28.3 ± 12.9 years (range, 11–66 years) (Table 1). A total of six patients were excluded owing to a prior history of MLKI surgery (*n* = 1), nonoperative management (*n* = 1), or incomplete medical records (*n* = 4). Of these patients, 75 patients (36%, 75/211) sustained pelvic and ipsilateral lower-extremity fractures. Incidence of fracture types varied based on fracture location, with TP/MLKI being present in 57% (43/75), FC/MLKI present in 47% (35/75), F/MLKI fractures present in 27% (20/75), P/MLKI present in 16% (12/75), TS/MLKI present in 9% (7/75), and FS present in 8% (6/75). Of the 75 patients with fractures, 17 (19%) were treated operatively and the remaining 58 patients with fractures (81%) were managed nonoperatively for their bony injuries. (Table 2).

**Table 1:** Demographics and characteristics of patients with and without fractures

	Non-fracture (*n* = 136)	Fracture (*n* = 75)	*P*-value
Age at surgery, years (SD)	27.2 (12.4)	30.2 (13.5)	0.106
BMI	28.7 (7.8)	28.4 (8.4)	0.822
Sex (male)	94 (69%)	53 (71%)	0.876
Mean days to surgery, d (SD)	146.5 (262)	103.5 (147)	0.193
High-energy mechanism	43 (29%)	57 (76%)	< 0.0001

^*^Independent *t*-tests were used to calculate the difference of means of age at surgery, BMI, and mean days to surgery between the fracture and non-fracture groups. The chi-squared test was used to determine no significant difference between sex of the groups. SD, standard deviation

**Table 2:** Demographics and characteristics of patients based on fracture location

	Tibial plateau	Femoral condyle	Tibial shaft	Femoral shaft	Fibula	Pelvis
Total (*n*)	43	34	7	6	20	12
Age, years (SD)	29.37 (14.31)	28.52 (12.9)	32.39 (11.6)	24.05 (6.95)	28.41 (12.17)	32.34 (15.95)
Sex (male) %	70	74	71	83	65	83
BMI (SD)	29.10 (9.8)	26.77 (7.04)	26.29 (5.13)	26.67 (4.07)	24.96 (6.76)	27.79 (7.40)
High-energy mechanism %	86	59	100	100	80	100
Average total fractures (SD)	1.81 (0.93)	1.91 (0.97)	1.71 (0.76)	3.17 (1.17)	2.15 (0.67)	1.50 (1.09)
Mean days to surgery (SD)	104 (153)	116 (162)	82 (54)	171 (164)	110 (171)	89 (51)

^*^Independent *t*-tests were used to calculate the difference of means of age at surgery, BMI, and mean days to surgery between the fracture and non-fracture groups. The chi-squared test was used to determine no significant difference between sex of the groups

Fracture patterns varied on the basis of MLKI ligamentous involvement. ACL-based MLKIs were the most frequently observed in fracture cases, accounting for 70% (43/75) of tibial plateau fractures, 68% (34/75) of femoral condyle fractures, 71% (7/75) of tibial shaft fractures, 50% (3/6) of femoral shaft fractures, 70% (14/20) of fibular fractures, and 67% (8/12) of pelvic fractures. Bicruciate-based MLKIs were identified in 50% (3/6) of femoral shaft fractures. The most common ligamentous pattern among fractures was ACL/MCL, which accounted for 35% (15/43) of tibial plateau fractures, 32% (11/34) of femoral condyle fractures, and 45% (9/20) of fibular fractures. ACL/LCL patterns were more commonly associated with tibial shaft fractures (29%; 2/7), femoral shaft fractures (50%; 3/6), and pelvic fractures (33%; 4/12). Two-ligament injuries remained the predominant MLKI pattern among fractures, comprising 74% (32/43) of tibial plateau injuries, 65% (22/34) of femoral condyle injuries, 71% (5/7) of tibial shaft injuries, 67% (4/6) of femoral shaft injuries, 70% (14/20) of fibular injuries, and 50% (6/12) of pelvic injuries.

The total number of fractures sustained per patient also varied by fracture type. Patients with FS/MLKI sustained an average of 3.17 total fractures (standard deviation [SD] 1.17) in the pelvis and ipsilateral lower extremity, the highest among all groups. Those with F/MLKI had 2.15 (SD 0.67) total fractures, FC/MLKI had 1.91 (SD 0.97), TP/MLKI had 1.81 (SD 0.93), TS/MLKI had 1.71 (SD 0.76), and P/MLKI had 1.5 (SD 1.09) total fractures on average (Table 3). High-energy mechanisms were more commonly associated with MLKIs in the fracture group compared with the non-fracture group (29% versus 76%; *p* < 0.001).

**Table 3:** Summary of the most frequent MLKI patterns and frequency of fractures requiring operative treatment for each fracture type observed

Fracture type	Total (*n*)	Cruciate-based pattern	Ligamentous pattern	Number-of-ligaments pattern	% operative treatment
Tibial plateau	43	ACL-based (70%)	ACL/MCL (35%)	Two-ligament (74%)	7
Femoral condyle	34	ACL-based (68%)	ACL/MCL (32%)	Two-ligament (65%)	18
Tibial shaft	7	ACL-based (71%)	ACL/MCL; ACL/LCL (29%)	Two-ligament (71%)	57
Femoral shaft	6	ACL; bicruciate-based (50%)	ACL/LCL (50%)	Two-ligament (67%)	100
Fibula	20	ACL-based (70%)	ACL/MCL (45%)	Two-ligament (70%)	5
Pelvic	12	ACL-based (67%)	ACL/LCL (33%)	Two-ligament (50%)	42

This table highlights the most common patterns observed for each category. Percentages are calculated independently from the total number of fractures for that specific location and are not intended to sum to 100% within each column. *ACL* anterior cruciate ligament, *PCL* posterior cruciate ligament, *MCL* medial collateral ligament, *LCL* lateral collateral ligament, *MLKI* multiligament knee injury

**Fig. 1:**
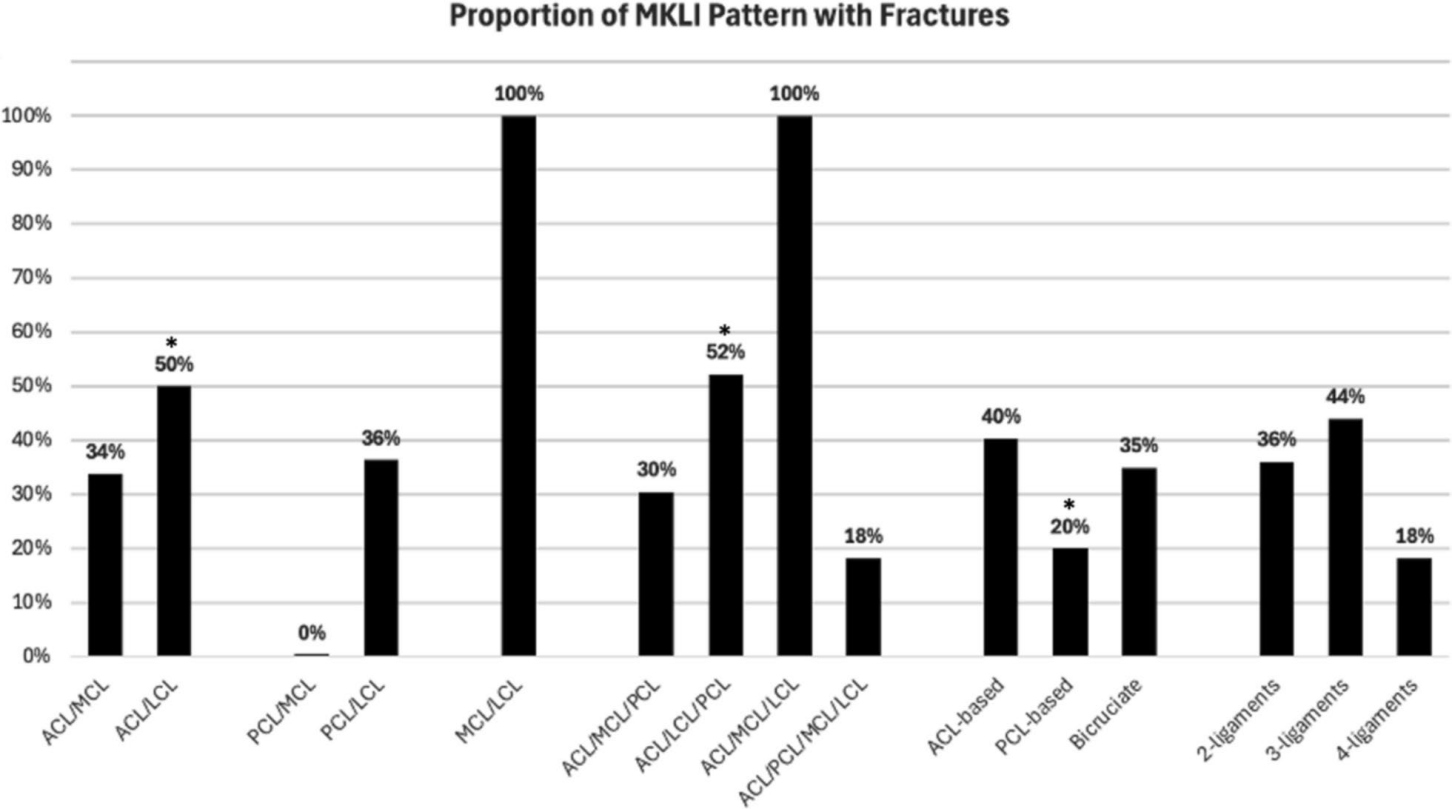
Proportion of MLKIs with a fracture. The *x*-axis of the figure categorizes different multiligament knee injury (MLKI) patterns on the basis of ligament involvement, including combinations of the anterior cruciate ligament (ACL), posterior cruciate ligament (PCL), medial collateral ligament (MCL), and lateral collateral ligament (LCL). In addition, MLKI patterns are grouped on the basis of ACL-based versus PCL-based injuries, bicruciate injuries, and the number of ligaments involved (two, three, or four). The *y*-axis represents the proportion of MLKIs associated with fractures, expressed as percentages ranging from 0% to 100%. An asterisk (^*^) indicates a statistically significant difference (*p* < 0.05) in the prevalence of that specific ligamentous pattern when comparing the fracture and non-fracture patient groups. *ACL* anterior cruciate ligament, *PCL* posterior cruciate ligament, *MCL* medial collateral ligament, *LCL* lateral collateral ligament, *MLKI* multiligament knee injury

Ligamentous injury patterns based on cruciate pattern revealed that ACL-based injuries were the most common, occurring in 65% (49/75) of fracture cases compared with 55% (75/136) in the non-fracture group (*p* = 0.150). ACL/MCL injuries were similar between fracture and non-fracture groups: 32% (24/75) versus 37% (50/136), respectively (*p* = 0.488). ACL/LCL injuries were significantly more common in the fracture group (29%; 22/75) versus the non-fracture group (18%; 24/136) (*p* = 0.049). PCL-based MLKIs were less frequent in the fracture group (4%; 3/75) compared with the non-fracture group (13%; 17/136) (*p* = 0.044). PCL/MCL injuries with fractures were absent in this cohort, compared with 2% (3/136) in the non-fracture group (*p* = 0.553). PCL/LCL injuries were similar between fracture and non-fracture groups: 5% (4/75) versus 5% (5/136), respectively (*p* = 1.00). Rates of bicruciate MLKIs with fractures (31%; 23/75) were similar to those without fractures (32%; 44/136) (*p* = 0.801). Bicruciate MLKIs with MCL (ACL/PCL/MCL) accounted for 9% (7/75) of patients with fractures, while accounting for 12% (16/136) of the non-fracture group (*p* = 0.588). Bicruciate MLKIs with LCL (ACL/PCL/LCL) occurred more frequently in the fracture group (5%, 4/75) compared with the non-fracture group (0%, 0/136) (*p* = 0.015) (Fig. 1). Two-ligament injuries were the most common overall, accounting for 71% (53/75) of all fracture cases, with no difference compared with the non-fracture group (71%; 97/136). Three-ligament injuries accounted for 29% (22/75) of the fracture group and 21% (28/136) (*p* = 0.177) of the non-fracture group. Four-ligament injuries account for 3% (2/75) of the fracture group and 7% (9/136) of the non-fracture group (*p* = 0.581) (Fig. 1).

## Discussion

In this retrospective analysis, we characterized the fracture burden and ligamentous injury patterns in MLKIs, with a particular focus on pelvic and ipsilateral lower-extremity fractures. Nearly one-third of patients sustained at least one fracture, with one-twelfth of patients sustaining fractures that required operative fixation. The majority of these concomitant fractures were therefore stable or minimally displaced and managed without surgical intervention. Patients with fractures were predominantly involved in high-energy mechanisms causing their MLKIs. Tibial plateau fractures were most common, followed by fractures of the femoral condyles, fibula, pelvis, tibial shaft, and femoral shaft. Notably, ACL/LCL injuries and bicruciate injuries involving the LCL were significantly more frequent among patients with fractures (as opposed to those without fractures), suggesting a predominance of varus stress and laterally directed force transmission through the knee. As hypothesized, PCL-based and ACL-based MLKIs differed in their fracture burden, with PCL-based injuries occurring less often in fracture patients and ACL-based injuries being common in patients with concomitant fracture. We rejected our secondary hypothesis in that concomitant fracture did not coincide with increasing number of ligaments injured when compared with a non-fracture cohort.

Fractures in knee injuries often present with significant complexity, involving both soft tissue and bony elements. The specific fracture patterns depend largely on knee position at the time of impact and the underlying mechanism of injury. Ipsilateral lower-extremity fractures are a common injury associated with individual knee ligament injuries, as well as MLKIs [8, 11, 12]. Among articular fractures, those involving the tibial plateau and femoral condyles have been extensively documented in the literature. Reported rates of tibial plateau fractures in association with knee ligamentous injuries have been reported to range from 40% to 77% [10, 13, 14]. In dislocated knees, fractures of the femoral condyles have been found to result in limited range of motion and significant functional deficits following fixation [15]. Furthermore, the presence of acute cortical fractures in ligamentous knee injuries often indicates concurrent soft tissue damage (e.g., meniscal tears), leading to inferior clinical outcomes [11]. While fracture patterns in single-ligament knee injuries have been well characterized, especially within the tibiofemoral joint [16, 17], there remains a paucity of data regarding the prevalence of ipsilateral lower-extremity fractures and their relationship to MLKI patterns.

The present study’s findings build upon the work of Becker et al. who documented a 58% prevalence of fractures in a cohort of 102 patients with MLKIs [10], notably higher than the 36% fracture rate in our series of 211 patients. This aligns with other studies indicating that ipsilateral lower-extremity fractures occur in 14–25% of MLKIs [18–20]. In contrast to the 25% tibial plateau fracture prevalence reported by Becker et al., our cohort's rate of 53% aligns more closely with other studies, which have documented prevalence rates ranging from 40% to 77% in similar patient populations [14, 21]. Compared with the cohort in the study conducted by Becker et al., this discrepancy also extends to femoral condyle fractures, which were also more common in our study. One plausible explanation for this discrepancy is that 75% of the injuries in their cohort were due to high-energy mechanisms, whereas only 47% (100/211) of our MLKIs resulted from high-energy trauma [10]. Furthermore, in our study, bicruciate MLKIs comprised only 24% (50/211) of the cohort, with a fracture rate of 52% in these patients. In contrast, Becker et al. reported that bicruciate MLKIs made up nearly 60% of their cohort, highlighting a notable difference in cohort composition between the studies. Their study, however, did not compare fracture rates across MLKI patterns. Our data suggest that patients with ACL/LCL and bicruciate with LCL MLKIs are more susceptible to ipsilateral lower-extremity fractures when compared with those of ACL/MCL and bicruciate with MCL MLKIs.

The clinical implications of these findings are significant for patient management and rehabilitation. The high prevalence of concomitant fractures sustained by more than one-third of patients with MLKI in our cohort necessitates a high index of suspicion during initial evaluation. The presence of a fracture, particularly those requiring operative fixation as seen in one-twelfth of our patients, invariably complicates postoperative care. Rehabilitation protocols must be modified to account for fracture stability, potentially involving delayed weight-bearing, altered range-of-motion limitations, and extended recovery timelines compared with isolated MLKI treatment. Furthermore, identifying high-risk ligamentous patterns, such as ACL/LCL injuries, can help surgeons anticipate the need for more complex surgical staging and counsel patients on the likelihood of a more challenging recovery. Understanding these injury patterns is a critical step toward optimizing rehabilitation strategies and managing patient expectations.

Several limitations of this study should be acknowledged. First, the retrospective, single-institution design may introduce selection bias and limit the generalizability of the findings to other populations and practice settings. Second, our results rely on the accuracy and completeness of radiographic and MRI documentation; inconsistencies in imaging protocols, timing, or quality across patients may have influenced both the identification of fractures and characterization of ligamentous injuries. Third, although we reported overall fracture occurrences, we did not differentiate specific fracture subtypes—such as lateral versus medial tibial plateau fractures—which could offer deeper insights into the relationship between fracture morphology and biomechanical forces leading to MLKIs. Fourth, the lack of standardized imaging protocols could have contributed to under- or overestimation of fracture rates, particularly if late or subtle fractures were missed. Furthermore, our cohort is limited to those with sufficiently documented imaging and clinical follow-up to identify additional surgeries associated with the indexed injury, potentially excluding patients with incomplete records or those treated elsewhere after initial evaluation. Additionally, our study is limited to those patients treated for MLKIs that involved two or more ligaments. This inherently excludes patients that may have been found to have an MLKI with a single ligament reconstruction with others that were treated non-operatively. Lastly, although we reported overall fracture occurrences, we did not differentiate specific fracture subtypes such as lateral versus medial tibial plateau fractures, which could offer deeper insights into the relationship between fracture morphology and biomechanical forces leading to MLKIs. Finally, our analysis is based on bivariate comparisons and does not include a multivariate regression model. While our study identifies significant associations between specific ligament injuries and fracture patterns, the lack of a multivariate analysis means we cannot definitively control for confounding variables or establish the independent predictive value of any single factor. These findings should be considered associative, and future studies with larger cohorts would be beneficial to build a formal predictive model. Fifth, we performed multiple statistical comparisons, which increases the possibility of a type I error, or a false-positive result. We did not apply a correction method, such as a Bonferroni adjustment, because our study was intended to be exploratory and hypothesis-generating. As such, findings with *p*-values approaching 0.05, such as the higher prevalence of ACL/LCL injuries in the fracture group, should be interpreted with caution until they can be validated by future research. Despite these limitations, we believe the findings of this study add meaningful insights into the body of research on MLKIs, highlighting key associations between fracture burden and ligamentous injury patterns that warrant further prospective, multicenter investigation.

## Conclusions

In this retrospective analysis of MLKIs, fractures were common, occurring in more than one-third of patients; however, only one-twelfth of which required operative intervention. Tibial plateau fractures were most common, followed by femoral condyle and pelvic fractures. ACL-based MLKIs were the most common ligament injury pattern observed concomitantly with an ipsilateral fracture. ACL/LCL and bicruciate-with-LCL patterns also commonly accompanied a fracture, suggesting a possible role of varus stress and laterally directed forces. Conversely, PCL-based MLKIs were less frequently associated with fractures. These findings underscore the need for heightened clinical suspicion of concomitant fractures in specific ligamentous patterns, which could facilitate improved preoperative planning and targeted surgical strategies.

## Data Availability

The deidentified datasets generated and/or analyzed during the current study are not publicly available in order to protect patient privacy, but they are available from the corresponding author, S.A., upon reasonable request and with institutional approval.

## Abbreviations

ACL: Anterior cruciate ligament
BMI: Body mass index
FC: Femoral condyle
F: Fibula
FS: Femoral shaft
IRB: Institutional Review Board
LE: Lower extremity
LCL: Lateral collateral ligament
MCL: Medial collateral ligament
MLKI: Multiligament knee injury
MRI: Magnetic resonance imaging
P: Pelvis
PCL: Posterior cruciate ligament
REDCap: Research Electronic Data Capture
SD: Standard deviation
SPSS: Statistical Package for the Social Sciences
TP: Tibial plateau
TS: Tibial shaft
